# Cervical strain elastography improves mid-trimester prediction of spontaneous preterm birth beyond cervical length: a single-center cohort study

**DOI:** 10.3389/fmed.2026.1863170

**Published:** 2026-06-17

**Authors:** Dongmei Zhang, Li Hou, Chunrong Li, Shiyue Peng, Liuying Zhou, Tongyong Luo

**Affiliations:** 1 Department of Ultrasound, Chengdu Women’s and Children’s Central Hospital, School of Medicine, University of Electronic Science and Technology of China, Chengdu, China; 2 Center for Pediatric Cardiology, Sichuan Provincial Women’s and Children’s Hospital, Affiliated Women’s and Children’s Hospital of Chengdu Medical College, Chengdu, Sichuan, China; 3 Department of Statistics, Columbia University, New York, NY, United States

**Keywords:** cervical length, cervix uteri, elastography, premature birth, prenatal ultrasonography, risk assessment

## Abstract

**Objective:**

Spontaneous preterm birth (sPTB) remains a leading cause of neonatal morbidity and mortality, and cervical length (CL) alone has limited predictive performance in asymptomatic women. Cervical strain elastography may capture biomechanical changes that precede cervical shortening. We aimed to evaluate the predictive value of mid-trimester cervical strain elastography parameters, alone and in combination with CL, for sPTB.

**Methods:**

In this single-center cohort study with prospectively collected data, 275 singleton pregnancies underwent transvaginal assessment at 18 + 0 to 23 + 6 weeks of gestation. Cervical strain elastography parameters, including hardness ratio (HR), internal os strain (IOS), external os strain (EOS), and the IOS/EOS ratio, were obtained using E-Cervix software, together with CL. The primary outcome was sPTB before 37 weeks. Logistic regression, receiver operating characteristic analysis, pairwise DeLong comparisons, and decision curve analysis were performed to assess predictive performance and clinical utility.

**Results:**

sPTB occurred in 37 of 275 women (13.5%). Among individual predictors, IOS showed the best discrimination for sPTB (AUC 0.797, 95% CI 0.715–0.878), followed by the IOS/EOS ratio (AUC 0.758, 95% CI 0.671–0.846). Among combined models, CL + IOS/EOS achieved the highest overall performance (AUC 0.828, 95% CI 0.771–0.885), with a sensitivity of 73% and a specificity of 82% at the Youden’s *J* optimal cut-off. Pairwise DeLong comparisons showed that CL + IOS/EOS significantly outperformed CL alone and IOS/EOS alone. Decision curve analysis suggested superior net benefit for the combined model across clinically relevant threshold probabilities.

**Conclusion:**

Mid-trimester cervical strain elastography, particularly IOS-related metrics, provides meaningful predictive information for sPTB. Combining elastography with CL improved discrimination and may enhance mid-trimester risk stratification beyond CL alone. External validation is warranted before routine clinical implementation.

## Introduction

Preterm birth (PTB), delivery before 37 + 0 weeks, remains the leading cause of death among children <5 years, and affected 13.4 million newborns, or 9.9% (≈1 in 10) of all live births worldwide in 2020 (1). Although China’s modeled PTB rate was only 6.1% (95% CI 5.1–7.4)—below the global average—the country still recorded about 0.75 million preterm births and ranked fourth globally in absolute numbers, after India, Pakistan and Nigeria (1). Although its etiology is multifactorial, premature cervical remodeling is the final common pathway to labor. During pregnancy, the cervix would progressively soften, shorten and dilate; when these biomechanical changes occur too early, spontaneous PTB (sPTB) follows (2).

Transvaginal sonographic measurement of cervical length (CL) is the current screening standard, but its predictive accuracy is modest. In a prospective Chinese cohort of 1,484 singleton pregnancies assessed at 11–13 + 6 weeks, first-trimester cervical length predicted sPTB <32 weeks with only fair discrimination: with an area under the ROC curve of 0.72 for a two-line measurement and 0.61 for the conventional single-line method (3). In a 9,410-participant United States cohort, a CL ≤ 25 mm between 22 and 30 weeks identified only 23% of women who later delivered preterm, with an AUC of 0.67 (4). These limitations have fueled interest in imaging techniques capable of quantifying cervical micro-mechanics rather than length alone.

Ultrasound elastography, either quasi-static strain imaging or shear-wave elastography, provides color-coded maps and quantitative parameters such as hardness ratio (HR), mean internal-os strain (IOS) and the IOS/EOS ratio. Early prospective work in low-risk women showed that a higher IOS during the second trimester (AUC 0.573) predicted sPTB when CL did not (5). Since then, the evidence base has expanded, especially in women with a prior spontaneous PTB (6). A 2024 single-center study of 71 asymptomatic women found that a red (soft) color code at the anterior internal os plus an lateral to the cervix ratio >0.72 predicted preterm delivery with 52.11% specificity, outperforming CL thresholds, Meta-analysis confirms pooled AUC ≈ 0.90 for elastography predictions (7, 8). Unlike shear-wave elastography, which measures absolute stiffness via Young’s modulus, strain elastography relies on relative tissue deformation and is operator-dependent, yet it remains effective for sPTB prediction (9). Real-time shear-wave elastography of 773 singleton pregnancies demonstrated that Young’s modulus at the internal os was an independent predictor of sPTB, and a multivariable model combining elastography with CL achieved an AUC of 0.98 (9). In progesterone-treated women with a short cervix (≤25 mm), lower HR and elevated IOS remained associated with sPTB despite therapy, illustrating the technique’s ability to identify residual risk (10). Most recently, a 2025 United States high-risk cohort with prior PTB showed that semi-automated E-Cervix metrics acquired at 18–23 weeks improved risk stratification compared with CL alone (6). Collectively, these data indicate that cervical stiffness metrics capture pathophysiological changes that precede sonographic shortening, yet heterogeneity in gestational age windows, imaging protocols and parameter selection still hampers clinical translation.

### Study rationale and objectives

We conducted a cohort study with prospectively collected data in a Chinese population to: (1) compare mid-trimester cervical elastography parameters (HR, IOS, EOS, IOS/EOS) and CL between women who experienced sPTB and those who delivered at term (FTB); and (2) evaluate the predictive performance of individual parameters and combined models (one elastography parameter + CL) for sPTB. We aimed to evaluate whether mid-trimester strain elastography parameters provide additional predictive information beyond cervical length and whether combined models may improve risk stratification for sPTB.

## Materials and methods

### Study design and setting

We performed a single-center cohort study in the Department of Obstetrics, Chengdu Women’s and Children’s Central Hospital, Chengdu, China, from 1 January 2022 to 31 December 2023. The study protocol was approved by the Institutional Ethics Committee (Approval No. 2021-94). All participants gave written informed consent. The study was reported in accordance with STROBE recommendations for cohort studies (11).

### Participants

Women with a viable singleton pregnancy who attended the routine second-trimester anomaly scan between 18 + 0 and 23 + 6 weeks were screened.

*Exclusion criteria as follows*: Multiple gestation; Known uterine malformation; Cervical or adjacent pelvic pathology that could compromise ultrasound assessment (e.g., cervical mass, cervical dilation at enrollment, placenta previa or vasa previa, or a large pelvic mass); Second-trimester pregnancy termination for any of the following reasons: major fetal structural or chromosomal abnormality, pre-viable preterm premature rupture of membranes (PPROM), hypertensive disorders of pregnancy, or placental abruption; lost to follow-up.

*Group-specific exclusions*: In the term-delivery group, women who had cervical cerclage, a cervical pessary, or progesterone therapy initiated before enrollment were excluded. In the preterm-delivery group, medically indicated PTB (e.g., due to pre-eclampsia or fetal growth restriction) was excluded; only spontaneous PTB cases were retained.

### Ultrasound acquisition

All scans were obtained transvaginally with a Samsung WS80A scanner (Samsung Medison, Seoul, Republic of Korea) and a 3–10 MHz endocavitary probe. The bladder was emptied and the patient was placed in lithotomy without external abdominal pressure. Scans were performed blinded to patient history, and images with motion artifacts >10% were discarded to ensure quality control. To minimize operator-dependent bias, standardized pressure was maintained by avoiding manual compression and relying on uterine arterial pulsation for tissue deformation.

#### Cervical length (CL)

CL was measured in the mid-sagittal plane according to ISUOG guidance, taking the mean of three consecutive images (12).

#### Strain elastography

Using the vendor’s E-Cervix software (version 3.5) on the same transvaginal ultrasound system. After a mid-sagittal cervical view was obtained, the probe was held steady without deliberate compression, and acquisition proceeded only when motion was stable. Tissue deformation was generated mainly by intrinsic maternal and vascular pulsation. The software automatically calculated HR, IOS, EOS, and the IOS/EOS ratio using the standardized 4-point method. Each parameter was measured three times and consistently averaged for analysis. Unlike stress-assisted quantitative systems, this study used conventional strain elastography to assess relative cervical deformation under a standardized protocol (13).

**Figure 1:**
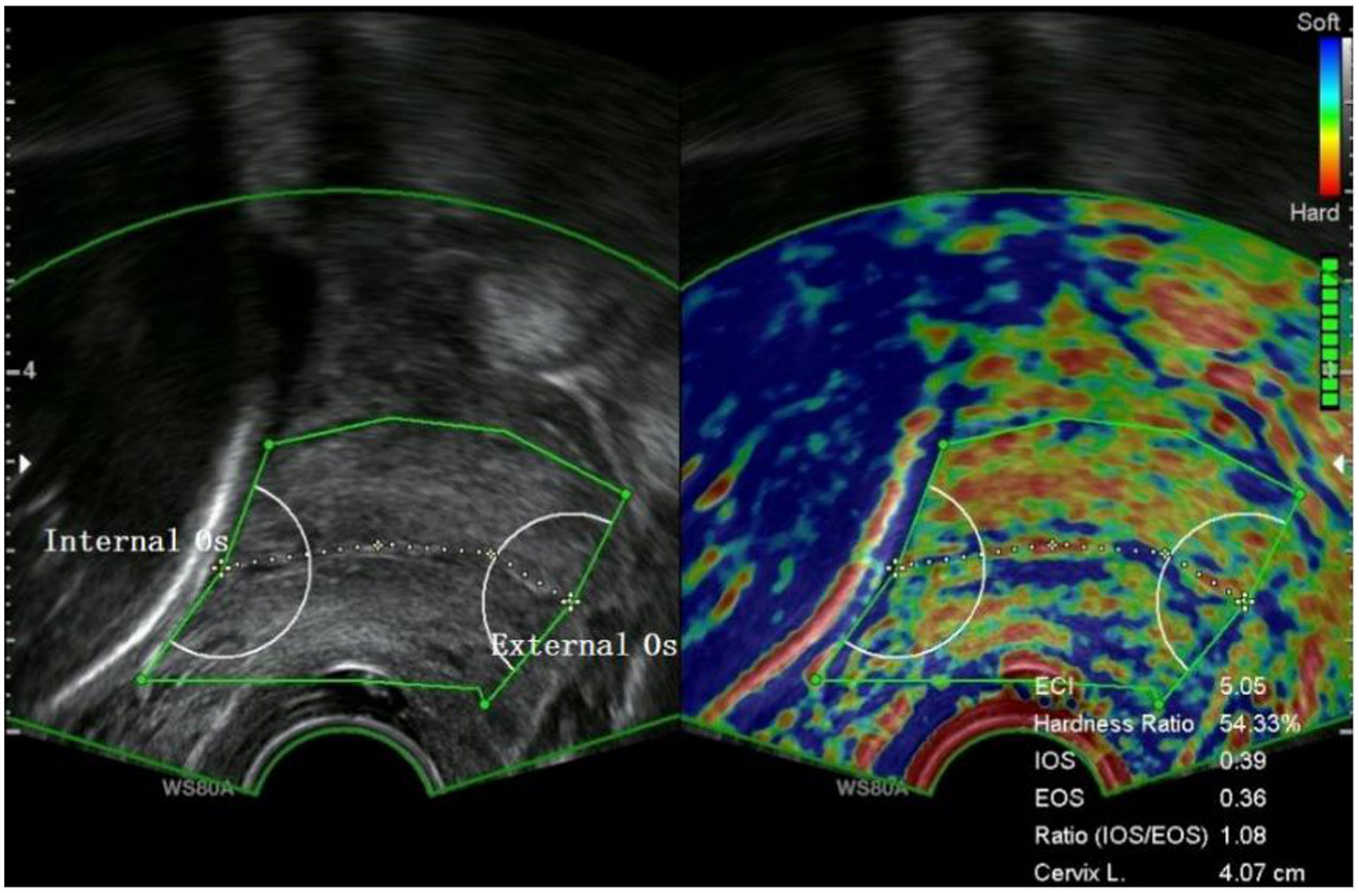
Transvaginal ultrasound elastography of the cervix showing measurement of elasticity parameters and cervical length.

The transvaginal cervical elastography workflow, including measurement of elasticity parameters (ECI, HR, IOS, EOS, IOS/EOS) and CL, is shown in Figure 1.

### Outcomes

The endpoint was sPTB <37^+0^ weeks, defined as spontaneous labor or preterm pre-labor rupture of membranes, excluding iatrogenic deliveries (14). The gestational age is calculated based on the crown-rump length (CRL) measured by ultrasound during the first trimester (between 8 and 13^+6^ weeks of pregnancy).

### Sample-size calculation

Sample-size calculation was performed using MedCalc software. Assuming the AUC of 0.80 for elastography parameters (vs. 0.70 for CL alone, based on prior studies (4)), with *α* = 0.05, power = 80%, and an expected event rate of 13% (based on published preterm birth rates from comparable tertiary maternal hospitals in our city, approximately 12.9% on average), the estimated sample size required was 250 participants.

### Statistical analysis

Normality of continuous variables was examined with the Shapiro–Wilk test. Data are presented as mean ± SD for normally distributed variables or median (inter-quartile range) when non-normal; between-group comparisons used the Student *t*-test or Mann–Whitney *U*-test, respectively. Categorical variables are reported as *n* (%) and were compared with the *χ*^2^ test or Fisher’s exact test.

#### Model building

For discrimination, we fitted logistic regression models to obtain predicted probabilities: (i) single-parameter models (each elastography parameter alone and CL alone); and (ii) combined models (CL plus one elastography parameter). To ensure comparability, combined-model analyses and decision-curve analyses (DCA) used a unified complete-case cohort (*N* = 275).

#### Discrimination (ROC/AUC) and statistical testing

We computed ROC curves and AUCs from predicted probabilities. 95% confidence intervals (CI) for AUCs were obtained using the DeLong method. For each combined model, we compared AUC against CL alone using DeLong’s test for correlated ROC curves (two-sided). A two-sided test was used as a conservative approach to assess differences in discrimination between correlated ROC curves.

#### Decision curve analysis (DCA)

Clinical utility was assessed by net benefit (NB) over threshold probabilities 0.10–0.30. NB was defined as NB = TP/*n* − FP/*n* × (pt/(1 − pt)). We benchmarked all models against Treat-All and Treat-None, and summarized mean NB (0.10–0.30), the maximum NB and corresponding threshold, the threshold range where NB exceeded both comparators, and the net reduction in interventions per 100 patients versus Treat-All.

All tests were two-sided with *α* = 0.05. To account for multiple comparisons of elastography parameters, *p*-values were adjusted using the Bonferroni correction where applicable. Statistics were performed in SPSS v26.0, MedCalc v20.218, and R v4.3.0 (packages pROC and rmda).

## Results

### Participant flow

**Figure 2:**
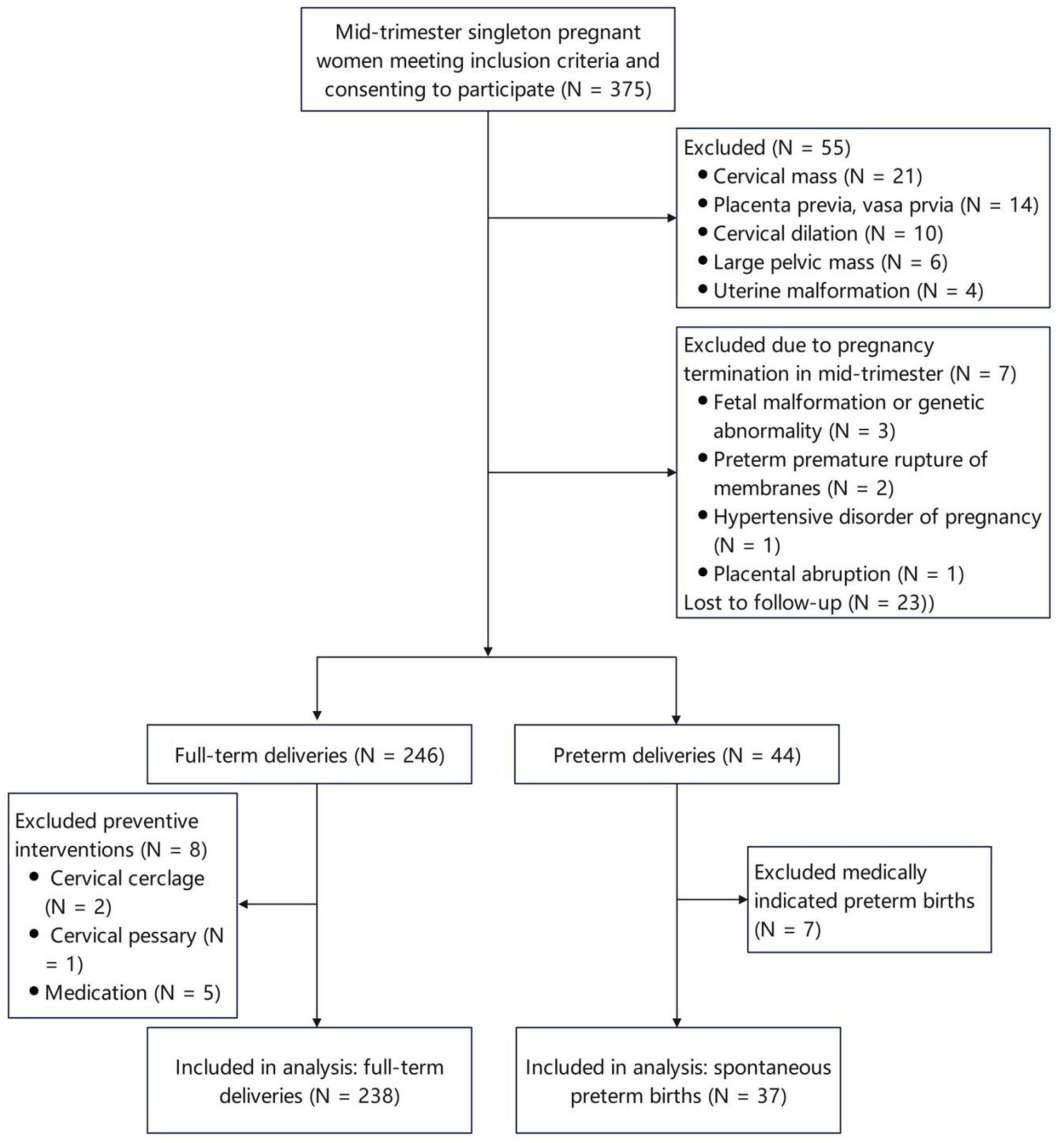
Flowchart of participant inclusion.

Of 375 women screened between 1 January 2022 and 31 December 2023, 100 met pre-specified exclusion criteria, leaving 275 for analysis (Figure 2). Follow-up was complete; 37 women (13.5%) experienced sPTB and 238 delivered at term.

### Baseline maternal characteristics

Maternal age, body-mass index, gravidity, prior pregnancy loss, use of assisted reproduction, and previous cervical surgery were comparable between groups (*p* > 0.05 for each; Table 1). The only expected difference was gestational age at delivery (median 33^+3^ weeks in the sPTB group vs. 39^+3^ weeks in controls, *p* < 0.001). Detailed baseline characteristics are summarized in Table 1.

**Table 1:** Baseline maternal characteristics of women with FTB and sPTB.

Characteristic	FTB (*n* = 238)	sPTB (*n* = 37)	*p*
Maternal age, y	29.00 (26.00–32.00)	31.00 (28.00–33.00)	0.700
BMI, kg/m^2^	24.80 (22.62–26.00)	24.84 (23.93–25.84)	0.531
Gravidity	2 (1–10)	2 (1–10)	0.596
Parity	64 (26.89%)	8 (21.62%)	0.498
History of mid-trimester abortion	25 (10.50%)	3 (8.11%)	0.654
IVF-ET	10 (4.20%)	2 (5.41%)	0.739
History of cervical conization	9 (3.78%)	2 (5.41%)	0.639
Gestational age at delivery, weeks	39^+3^ (38^+1^–39^+4^)	33^+3^ (31^+1^–36^+2^)	<0.001

### Cervical elastography and length

In mid-trimester, CL was significantly shorter in the sPTB group than in controls (median 2.83 cm [IQR 1.88–3.50] vs. 3.73 cm [3.20–4.26]; *p* < 0.001). Strain elastography indicated a softer cervix in sPTB: HR 50.0% vs. 66.1% (*p* < 0.001), IOS 0.38 vs. 0.25 (p < 0.001), and IOS/EOS 1.20 vs. 0.83 (*p* < 0.001). EOS and ECI did not differ significantly (*p* = 0.080 and *p* = 0.051, respectively).

**Figure 3:**
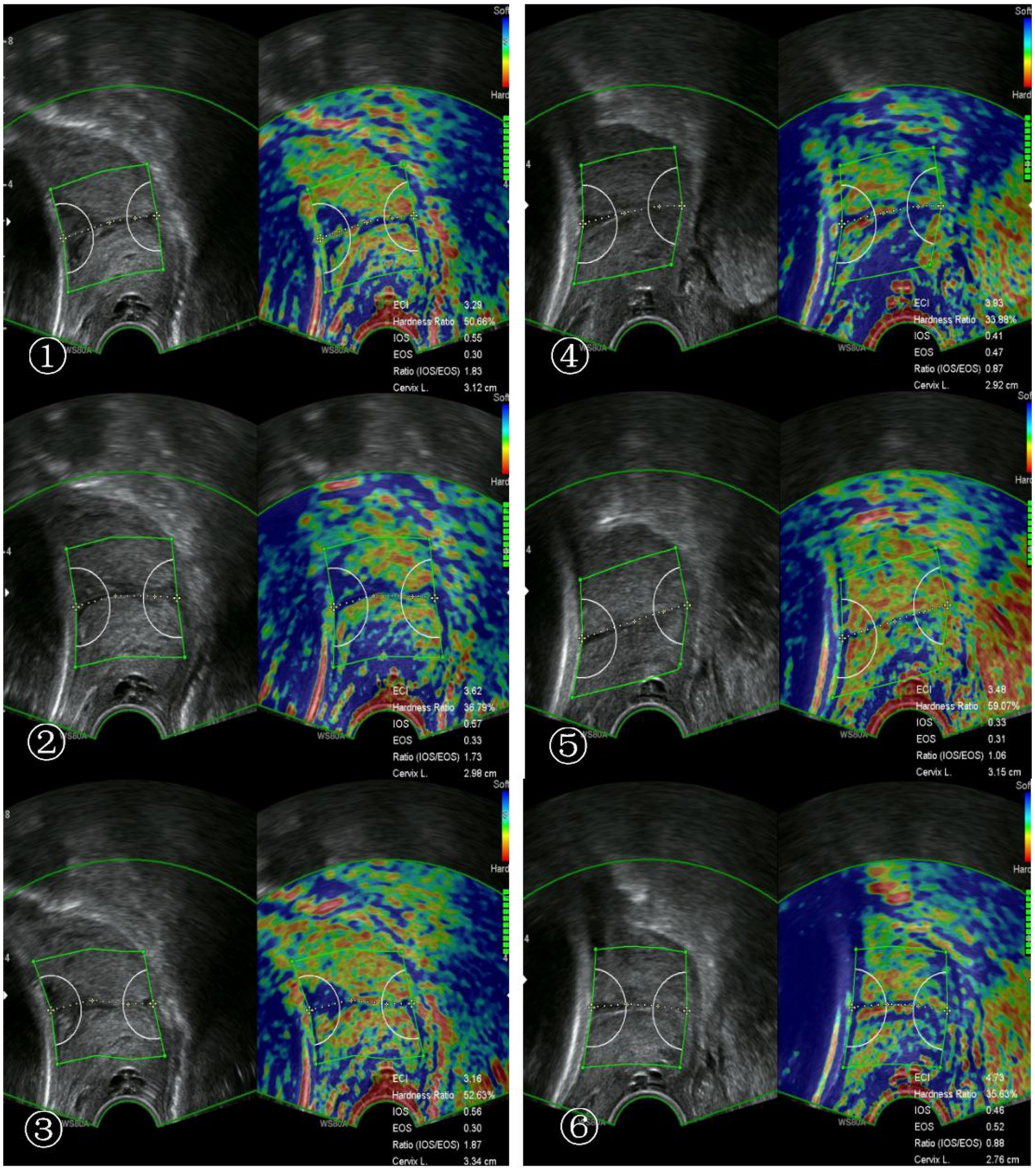
Representative elastograms in sPTB (panels ①–③, 29 years, 32 + 2 weeks) and FTB (panels ④–⑥, 31 years, 34 + 3 weeks).

The representative elastograms from sPTB and FTB women, highlighting softer cervices in the sPTB group were displayed in Figure 3. The statistical comparisons of elastography parameters and cervical length, confirming significant differences in HR, IOS, IOS/EOS and CL, can be seen in Table 2.

**Table 2:** Comparison of mid-trimester cervical elastography parameters and cervical length in sPTB vs. FTB.

Metrics	FTB group (*n* = 238)	sPTB group (*n* = 37)	*p*
Gestational age at ultrasound, weeks	21^+6^ (17^+5^, 25^+4^)	21^+3^ (18^+5^, 26^+0^)	0.661
ECI	3.87 (3.26–4.65)	4.35 (3.28–5.56)	0.051
HR (%)	66.07 (55.90–76.14)	50.00 (37.43–59.63)	<0.001
IOS	0.25 (0.19–0.30)	0.38 (0.29–0.48)	<0.001
EOS	0.31 (0.25–0.38)	0.33 (0.30–0.42)	0.080
IOS/EOS	0.83 (0.65–1.04)	1.20 (0.94–1.36)	<0.001
CL (cm)	3.73 (3.20–4.26)	2.83 (1.88–3.50)	<0.001

### Discrimination and threshold performance

**Figure 4:**
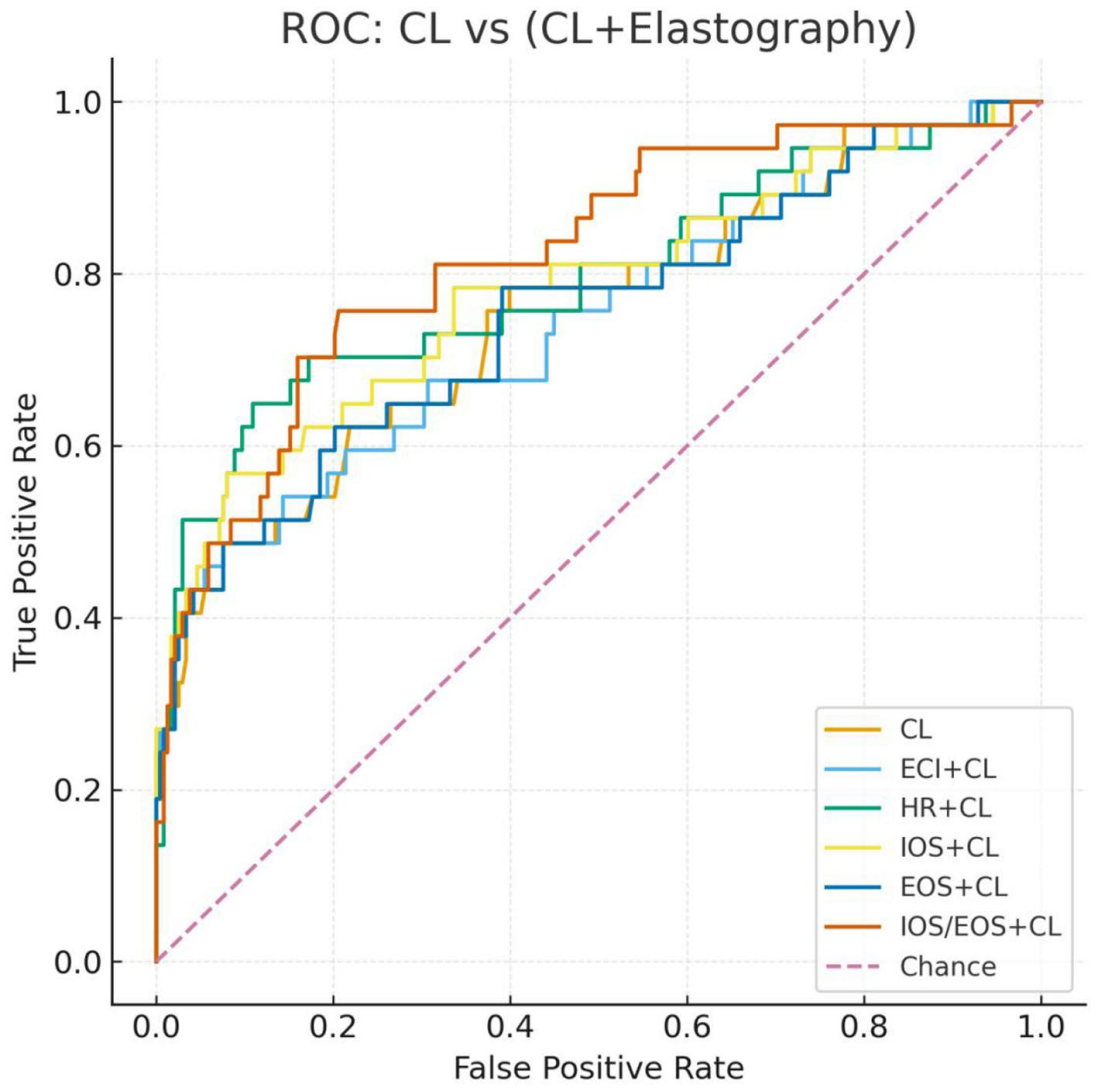
ROC curves for CL and combined models (CL + ECI, CL + HR, CL + IOS, CL + EOS, and CL + IOS/EOS).

Among single predictors, IOS demonstrated the strongest individual discrimination with an AUC of 0.797 (95% CI 0.715–0.878), followed by IOS/EOS 0.758 (0.671–0.846), CL 0.752 (0.657–0.847), and HR 0.752 (0.662–0.842); ECI and EOS yielded lower accuracy (0.600 [0.483–0.716] and 0.590 [0.488–0.691], respectively). The ROC curves for CL and the combined models are shown in Figure 4, and the full diagnostic performance metrics of the single and combined models at the Youden’s *J* optimal cut-off are summarized in Table 3. At this threshold, CL + IOS/EOS provided the best sensitivity–specificity balance, whereas CL + IOS and CL + HR favored higher specificity and more favorable positive likelihood ratios.

**Table 3:** Diagnostic performance of single and combined models at Youden’s *J* optimal cut-off.

Model	AUC (95% CI)	Optimal cut-off	Sensitivity (%)	Specificity (%)	PPV (%)	NPV (%)	+LR	−LR
**IOS**	**0.797 (0.715–0.878)**	**0.29**	**75.68**	**71.43**	**29.2**	**95**	**71.43**	**0.34**
IOS/EOS	0.758 (0.671–0.846)	1.13	62.16	83.19	36.5	93.4	3.7	0.45
HR	0.752 (0.662–0.842)	2.64	48.65	92.44	50	92.1	6.43	0.56
ECI	0.600 (0.483–0.716)	4.89	43.24	82.35	27.6	90.3	2.45	0.69
EOS	0.590 (0.488–0.691)	0.3	75.68	50	19	93	1.51	0.49
**CL**	**0.752 (0.657–0.847)**	**52.76**	**64.86**	**81.51**	**35.3**	**93.7**	**3.51**	**0.43**
**CL+IOS/EOS**	**0.822 (0.743–0.901)**	**—**	**75.68**	**79.41**	**36.36**	**95.45**	**3.68**	**0.31**
CL + HR	0.792 (0.698–0.886)	**—**	64.86	89.08	48	94.22	5.94	0.39
CL + IOS	0.782 (0.688–0.875)	**—**	56.76	92.02	52.5	93.19	7.11	0.47
CL + EOS	0.751 (0.654–0.848)	**—**	62.16	79.83	32.39	93.14	3.08	0.47
CL + ECI	0.744 (0.647–0.842)	**—**	48.65	92.44	50	92.05	6.43	0.56

The bold font displays the diagnostic performance values of CL model,the max AUCs in single, combined models at the Youden’s J optimal cut-off.

Pairwise DeLong comparisons of combined models vs. CL alone showed that CL + IOS/EOS improved discrimination significantly (AUC 0.822 vs. 0.752; ΔAUC = +0.070; *p* = 0.005) and that CL + IOS was also significantly superior to CL (0.782 vs. 0.752; ΔAUC = +0.030; *p* = 0.002). CL + HR showed a numerical increase (0.792 vs. 0.752; ΔAUC = +0.040) but did not reach conventional significance (*p* = 0.056); CL + EOS and CL + ECI did not improve upon CL (Table 4).

**Table 4:** DeLong comparisons: combined models vs. CL alone.

Model compaison	AUC_combined (95% CI)	ΔAUC	*p* (DeLong)
CL + IOS/EOS vs. CL	0.822 (0.743–0.901)	0.070	0.005
CL + HR vs. CL	0.792 (0.698–0.886)	0.040	0.056
CL + IOS vs. CL	0.782 (0.688–0.875)	0.030	0.002
CL + EOS vs. CL	0.751 (0.654–0.848)	−0.001	0.735
CL + ECI vs. CL	0.744 (0.647–0.842)	−0.008	0.396

### Decision-curve analysis (DCA): net benefit over 0.10–0.30

**Figure 5:**
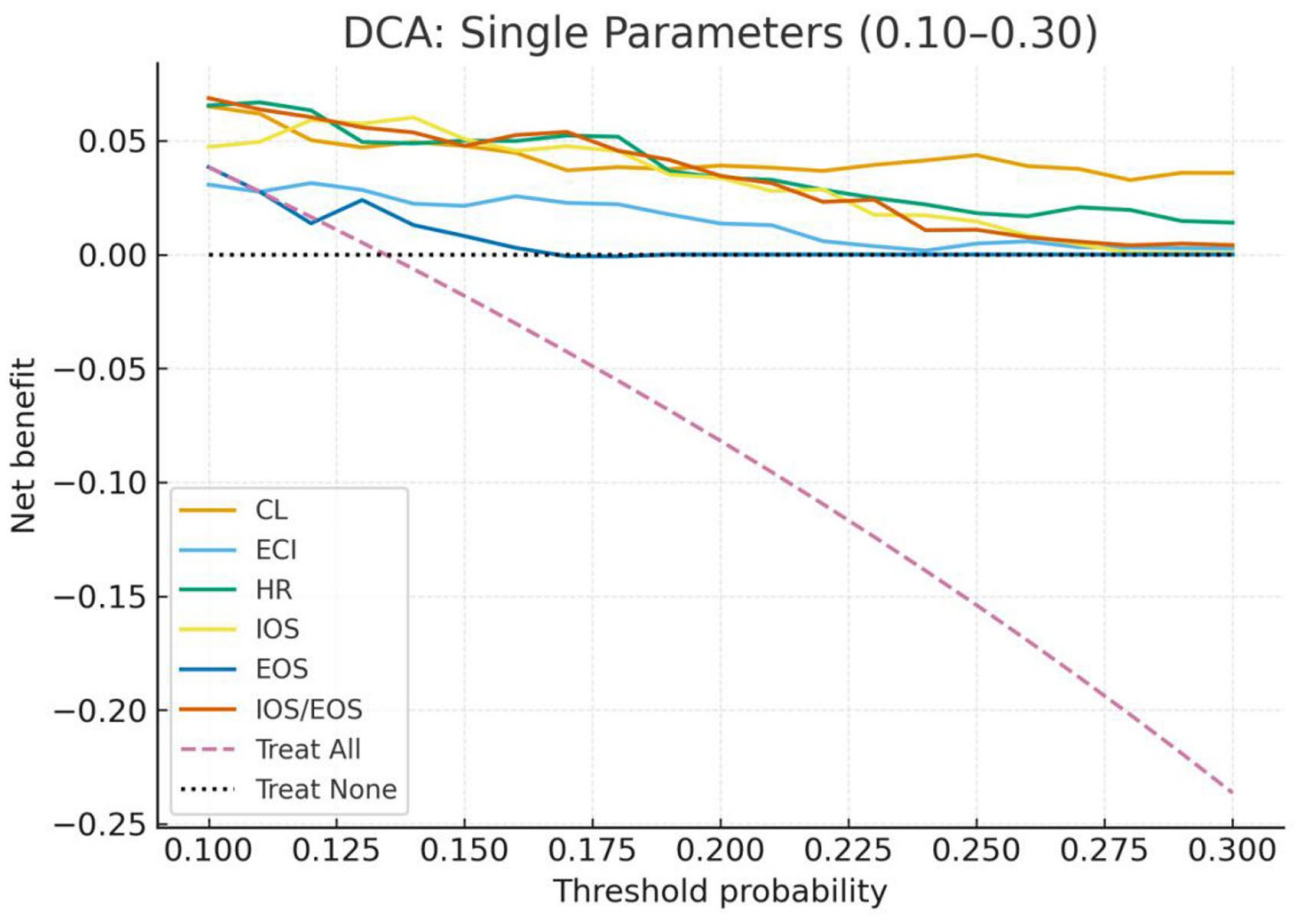
Decision curves for single-parameter models (thresholds 0.10–0.30).

**Figure 6:**
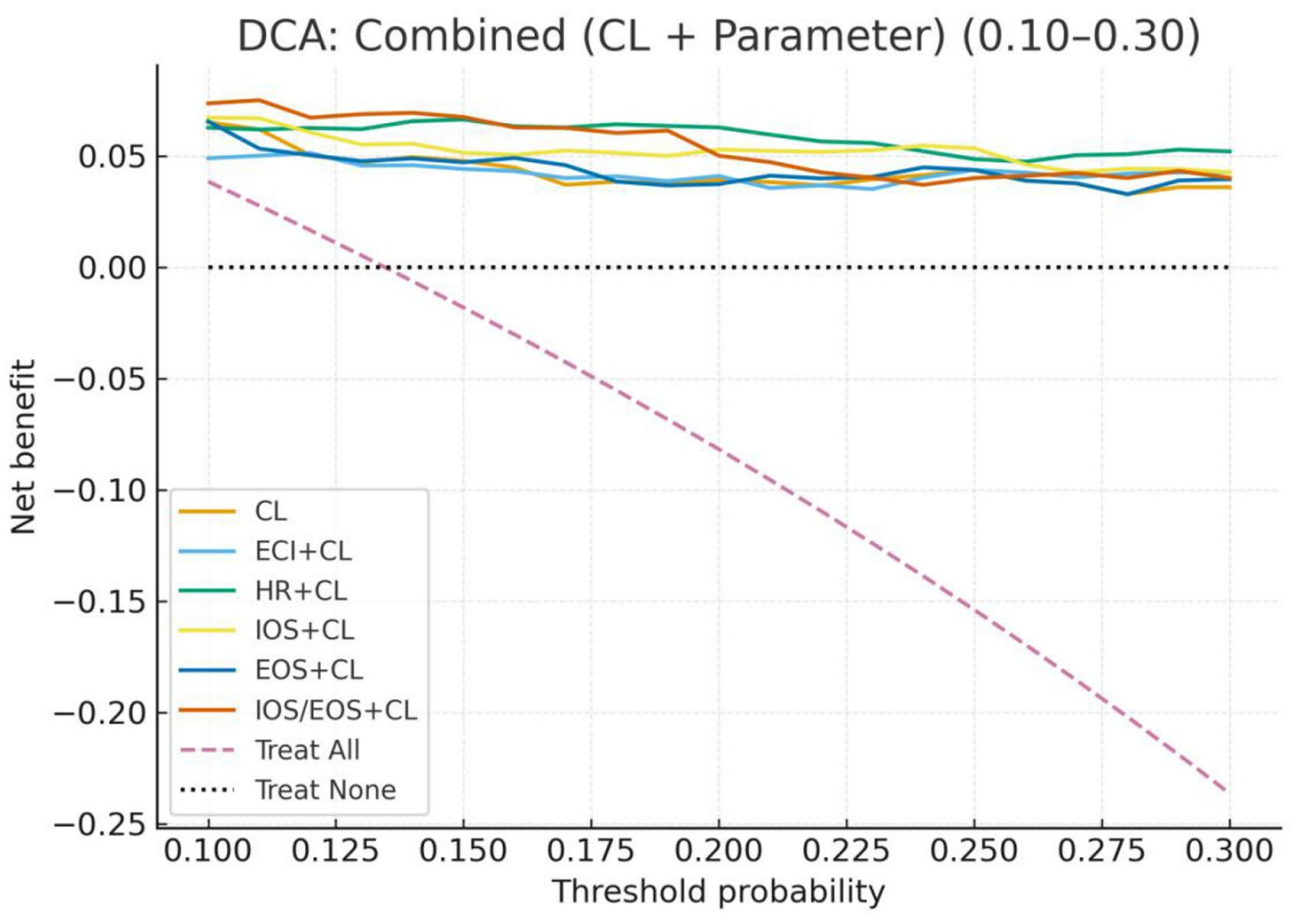
Decision curves for combined models (CL + elastography; thresholds 0.10–0.30).

Across clinically relevant thresholds (10–30%), all combined models outperformed Treat-All and Treat-None, and exceeded CL alone (Figures 5, 6). Averaged across the interval, CL + HR achieved the highest mean net benefit (0.058), followed by CL + IOS/EOS 0.054 and CL + IOS 0.052; for reference, CL alone averaged 0.042. The largest maximum NB was observed for CL + IOS/EOS (0.075 at pt. ≈ 0.11). Each combined model showed a broad superiority range where its NB exceeded both comparators (details in Table 5).

**Table 5:** Decision-curve analysis (DCA) summary across threshold probabilities 0.10–0.30.

Combined model	Single model						Combined model (CL+)				
	CL	ECI	HR	IOS	EOS	IOS/EOS	ECI	HR	IOS	EOS	IOS/EOS
Mean NB	0.042	0.015	0.037	0.031	0.005	0.033	0.042	0.058	0.052	0.043	0.054
Max NB	0.065	0.031	0.067	0.06	0.038	0.069	0.051	0.066	0.067	0.065	0.075
Best threshold	0.1	0.12	0.11	0.14	0.1	0.1	0.12	0.15	0.1	0.1	0.11

For clinical translation, the net reduction in unnecessary interventions per 100 patients versus Treat-All was as follows:

$$\begin{array}{c} \begin{array}{l} \mathrm{pt}=0.10:\mathrm{CL}+\mathrm{IOS}/\mathrm{EOS}\approx 31.6,\mathrm{CL}+\mathrm{IOS}\approx 25.8,\\ \mathrm{CL}\approx 24.0,\mathrm{CL}+\mathrm{HR}\approx 21.8 \end{array}\\ \begin{array}{l} \mathrm{pt}=0.20:\mathrm{CL}+\mathrm{HR}\approx 57.8,\mathrm{CL}+\mathrm{IOS}\approx 53.8,\\ \mathrm{CL}+\mathrm{IOS}/\mathrm{EOS}\approx 52.7,\mathrm{CL}\approx 48.4 \end{array}\\ \begin{array}{l} \mathrm{pt}=0.30:\mathrm{CL}+\mathrm{HR}\approx 67.3,\mathrm{CL}+\mathrm{IOS}\approx 65.1,\\ \mathrm{CL}+\mathrm{IOS}/\mathrm{EOS}\approx 64.5,\mathrm{CL}\approx 63.5 \end{array} \end{array}$$

CL + IOS/EOS offered the strongest utility at lower decision thresholds (approximately pt. 0.10–0.15) and the largest peak NB, whereas CL + HR delivered the highest average NB across 0.10–0.30. Taken together with the DeLong results, CL + IOS/EOS and CL + HR appear to be the most promising combinations.

## 4 Discussion

### Principal findings

In a mid-trimester cohort of 275 pregnancies (sPTB 13.5%), we found that elastography-derived indices provide incremental value beyond CL for risk stratification of sPTB. Among single parameters, IOS achieved the highest discrimination (AUC 0.797), and adding elastographic information to CL improved model performance. These findings are broadly consistent with prior studies and add further evidence that cervical biomechanical markers may provide information beyond CL for sPTB risk stratification (15–18). In paired DeLong comparisons, CL + IOS/EOS (AUC 0.822; ΔAUC +0.070; *p* = 0.005) and CL + IOS (AUC 0.782; ΔAUC +0.030; *p* = 0.002) were significantly superior to CL alone (AUC 0.752). DCA over clinically relevant thresholds (pt = 0.10–0.30) showed that all combined models offered higher net benefit than CL alone; CL + HR yielded the highest average net benefit across the interval, whereas CL + IOS/EOS achieved the largest maximum net benefit (best pt. ≈ 0.11).

Taken together, the AUC gains for CL + IOS/EOS (and CL + IOS) and the sustained NB advantage of CL + HR and CL + IOS/EOS across 0.10–0.30 suggest that biomechanical indices may provide incremental value beyond CL for mid-trimester sPTB risk stratification, although the magnitude of improvement was moderate.

### Interpretation in context of cervical biomechanics

Elastography captures cervical tissue deformability, which plausibly changes in the trajectory toward sPTB (4, 9). IOS, reflecting strain near the internal os, may be particularly sensitive to biomechanical remodeling at the site where funneling and early effacement often begin; this aligns with prior reports that higher IOS signals increased risk (2, 15). Regional mapping focused on the internal os has shown moderate discrimination and site-specific differences, consistent with our observation that IOS and IOS/EOS carry complementary signal to CL (19). The IOS/EOS ratio summarizes a cranio-caudal gradient in strain; its added value with CL suggests that spatial heterogeneity in cervical stiffness contains clinically relevant information not fully represented by a single linear length measurement (18). By contrast, HR showed a profile suggestive of higher specificity, which may explain its stronger average clinical utility in DCA despite a non-significant AUC gain versus CL. Beyond mechanics, inflammation and microbiome-mediated pathways have been implicated in cervical remodeling, providing biological plausibility for the observed elastographic changes (2, 20).

### Clinical implications and decision thresholds

These findings support integrating elastography + CL into mid-trimester assessment when intervention thresholds lie within 10–30% predicted risk, where preventive strategies and intensified surveillance are often considered. DCA quantifies these trade-offs: at pt. = 0.10, CL + IOS/EOS could avert ~32 unnecessary interventions per 100 patients versus Treat-All while maintaining high NPV; at pt. = 0.20–0.30, CL + HR shows the most consistent gains, aligning with care policies that prioritize fewer false positives (e.g., resource-constrained settings or when downstream therapies carry non-trivial burdens) (17). Methodological advances in acquisition and post-processing (e.g., higher spatial resolution, improved stability metrics, quantitative stiffness estimation) may further enhance the clinical signal of elastography, particularly when combined with CL and clear threshold policies (21). These findings should be interpreted as suggesting possible incremental utility for risk stratification within selected threshold ranges, rather than immediate readiness for routine clinical use.

### Comparison with prior evidence

Prior work has shown that CL alone incompletely captures biomechanical readiness of the cervix and that elastographic indices correlate with subsequent sPTB risk (18). Our findings reinforce and extend this evidence by demonstrating paired, DeLong-tested gains in discrimination when elastographic information is added to CL and by translating those gains into decision-level benefit across clinically pertinent thresholds. The present results are directionally consistent with reports across low- and high-risk cohorts and with studies focusing on internal-os–centric measures, supporting further evaluation of biomechanical assessment alongside CL in future validation studies (16, 17). The distinct profiles of CL + IOS/EOS (strong maximal gain at lower pt) and CL + HR (highest mean benefit across 0.10–0.30) may help reconcile mixed findings in the literature by emphasizing which threshold policies each parameter best supports.

### Limitations

First, models were evaluated with internal discrimination; external validation and calibration (e.g., calibration slope, Brier score) were not reported and should precede clinical deployment. Second, elastography is operator- and device-dependent; multicenter standardization of acquisition and post-processing is essential for generalization, particularly as newer platforms improve spatial/quantitative fidelity (22). In addition, true acquisition-level reproducibility of cervical strain elastography was not assessed in this study, because repeat measurements were performed offline on stored images rather than through repeated image acquisition. Third, complete-case analysis may introduce selection bias if missingness is not completely at random.

## Conclusion

In conclusion, this prospective cohort study suggests that mid-trimester cervical strain elastography, particularly IOS-related parameters, can provide clinically relevant information for identifying women at increased risk of spontaneous preterm birth. Among the evaluated models, the combination of CL and IOS/EOS achieved the best overall predictive performance, supporting the view that cervical biomechanics may complement rather than replace cervical length in risk assessment. These findings should, however, be interpreted with caution given the single-center design and the lack of external validation. Larger multicenter studies are needed to externally validate these models and assess their calibration and generalizability across different populations, and to determine whether incorporating cervical elastography into prenatal surveillance pathways can improve maternal and neonatal outcomes.

## Data Availability

The raw data supporting the conclusions of this article will be made available by the authors, without undue reservation.
